# Predicting Childhood Obesity Based on Single and Multiple Well-Child Visit Data Using Machine Learning Classifiers

**DOI:** 10.3390/s23020759

**Published:** 2023-01-09

**Authors:** Pritom Kumar Mondal, Kamrul H. Foysal, Bryan A. Norman, Lisaann S. Gittner

**Affiliations:** 1 Department of Industrial, Manufacturing & Systems Engineering, Texas Tech University, Lubbock, TX 79409, USA; 2 Department of Electrical and Computer Engineering, Texas Tech University, Lubbock, TX 79409, USA; 3 Department of Public Health, Texas Tech University Health Sciences Center, Lubbock, TX 79430, USA

**Keywords:** childhood obesity, machine learning, classification, BMI, well-child visit

## Abstract

Childhood obesity is a public health concern in the United States. Consequences of childhood obesity include metabolic disease and heart, lung, kidney, and other health-related comorbidities. Therefore, the early determination of obesity risk is needed and predicting the trend of a child’s body mass index (BMI) at an early age is crucial. Early identification of obesity can lead to early prevention. Multiple methods have been tested and evaluated to assess obesity trends in children. Available growth charts help determine a child’s current obesity level but do not predict future obesity risk. The present methods of predicting obesity include regression analysis and machine learning-based classifications and risk factor (threshold)-based categorizations based on specific criteria. All the present techniques, especially current machine learning-based methods, require longitudinal data and information on a large number of variables related to a child’s growth (e.g., socioeconomic, family-related factors) in order to predict future obesity-risk. In this paper, we propose three different techniques for three different scenarios to predict childhood obesity based on machine learning approaches and apply them to real data. Our proposed methods predict obesity for children at five years of age using the following three data sets: (1) a single well-child visit, (2) multiple well-child visits under the age of two, and (3) multiple random well-child visits under the age of five. Our models are especially important for situations where only the current patient information is available rather than having multiple data points from regular spaced well-child visits. Our models predict obesity using basic information such as birth BMI, gestational age, BMI measures from well-child visits, and gender. Our models can predict a child’s obesity category (normal, overweight, or obese) at five years of age with an accuracy of 89%, 77%, and 89%, for the three application scenarios, respectively. Therefore, our proposed models can assist healthcare professionals by acting as a decision support tool to aid in predicting childhood obesity early in order to reduce obesity-related complications, and in turn, improve healthcare.

## 1. Introduction

Childhood obesity has become a pressing public health problem [1,2]. Studies have found that the incidence of childhood obesity has tripled in recent years [3]. Obesity is a major lifestyle disease leading to serious comorbidities [4]. The World Health Organization (WHO) declared that, by 2030, 30% of deaths will be caused by preventable lifestyle-related illnesses [5]. Early preventive strategies are paramount, because children with obesity are at risk for serious lifestyle-related illnesses in adulthood [6]; therefore, determining the obesity trends of children in early life can potentially prevent future health comorbidities and extend their lives.

There is a need for childhood obesity risk prediction models in order to facilitate early interventions [7,8]. Therefore, an accurate and usable prediction model that only requires limited data is needed. In this study, we present several machine learning (ML)-based approaches to determine obesity trends in children based on real data acquired from a Midwestern health maintenance organization. We formulated different ML models to detect childhood obesity trends before age five based on single and multiple data from well-child visits. Predicting whether a child is going to be obese at age 5 is important, because 50% of children with obesity aged between 6 to 9 years and 80% of adolescents with obesity aged between 10 to 14 years are at risk of becoming adults with obesity, whereas only 25% of children with obesity aged less than 5 years are at risk of being obese when they become adults [9,10].

The World Health Organization (WHO) and the Centers for Disease Control and Prevention (CDC) growth charts are used to determine the current obesity level (normal weight, overweight, or obese) of a child based on the child’s current BMI value. These kinds of charts only identify the child’s present obesity level and, therefore, cannot be used to predict the future obesity category of a child, i.e., cannot predict the obesity level at age five of a currently two-year-old child. This highlights the need to develop data-driven ML models that use retrospective data to predict a child’s future obesity category.

Previous research to predict childhood obesity used factors such as: race, age of walking, solid food introduction age, breastfeeding duration, solid food types, dietary pattern of the family, family obesity history, family environment, socioeconomic status, and medication. However, many parents are reluctant to share this type of information because they resist the identification of childhood obesity in their child. Thus, an alternative approach based only on anthropomorphic measurements rather than family factors is needed [11,12]; moreover, often it is difficult to obtain reliable family factors. Therefore, the proposed methods predict whether a child will be normal weight, overweight, or obese at age five, using basic information such as gender, birth BMI, birth height (height is widely referred to as length for children below 2 years of age), birth weight, gestational age, and BMI on different days since birth (DSBs).

One of the biggest challenges regarding childhood obesity prediction is the lack of growth data, because well-child visits do not occur on the exact suggested schedule and some of these visits may not occur at all due to financial and other socioeconomic conditions [13,14]. Sometimes, only the current BMI information may be available, meaning no information related to previous well-child visits is available. Additionally, it may be necessary to make predictions based on only early well-child visit data [15]. Therefore, we propose different models for childhood obesity prediction, considering such application scenarios, and develop models that predict childhood obesity based on a single well-child visit, multiple early well-child visits, and multiple random well-child visits data. The remainder of this paper is organized as follows: we provide a literature review, followed by our proposed methodology, followed by results, and then discussion and conclusions.

## 2. Literature Review

Present methods for childhood obesity prediction can be broadly categorized into three types, namely risk factor (threshold)-based categorizations, regression analysis, and machine learning-based classifications. Risk factor-based obesity prediction relies on specific factors, such as biological, community, family, and environmental factors. These factors include fetal growth, maternal physical activity, and maternal tobacco use, among others [16]. The effectiveness of risk factor-based approaches is highly subjective because it depends on the correctness of scoring a child with respect to the different risk factors. The data driven approaches correspond to automatic and more accurate predictions. Multiple linear regression models rely on longitudinal and cross-sectional information, i.e., change in the feature values over time for an individual subject and the differences in the feature values across all the subjects [17]. However, there is no guarantee that the model can accurately predict the inter-subject variability coefficient, especially when the data size is limited and the level of variance changes over time [18]. The biggest strength of machine learning models is the capability of discovering hidden trends and patterns in data. Colmenarejo discussed the advantages and disadvantages of both ML and statistical methods and suggested using ML methods to have a higher prediction accuracy [19]. ML algorithms offer sophisticated and powerful techniques for analyzing critical factors to predict obesity risks and outcomes [6].

Over the years, ML algorithms have proven very useful in predicting obesity for older children, adolescents, and adults. Dunstan et al. used support vector machine, random forest, and extreme gradient boosting algorithms to predict nationwide obesity prevalence by using food and beverage sales-related data and found that baked goods and flours, cheese, and carbonated drinks are more relevant food types for obesity prediction [20]. Thamrin et al. developed three ML algorithms (naïve Bayes, classification and regression trees (CART), and logistic regression) to predict obesity in adults by using publicly available data and compared the performance of these three algorithms [21]. Cheng et al. used 11 classification algorithms (logistic regression, radial basis function (RBF), naïve Bayes, classification via regression (CVR), local *k*-nearest neighbors (*k*-NN), a decision table, random subspace, random tree, a multi-objective evolutionary fuzzy classifier, and a multilayer perceptron) to predict obesity in adults and achieved a highest overall accuracy of 70% with a random subspace algorithm [22]. Cervantes et al. developed decision tree (DT), *k*-means, and support vector machine (SVM)-based data mining techniques to identify obesity levels among young adults between 18 and 25 years of age so that interventions could be undertaken to maintain a healthier lifestyle in the future [23]. Gupta et al. developed a deep learning model (long short-term memory (LSTM)), which predicted obesity between 3 and 20 years of age with 80% accuracy using unaugmented electronic health record (EHR) data from 1 to 3 years prior [24]. Marcos-Pasero used random forest (RF) and gradient boosting to predict the BMI from 190 multidomain variables (data collected from 221 children aged 6 to 9 years) and determined the relative importance of the predictors [25]. Zare et al. used kindergarten-level BMI information, demographic, socioeconomic information such as family income, poverty level, race, ethnic compositing, housing, parent education, and family structure to predict obesity at the fourth grade and achieved an accuracy of about 87% by using logistic regression and an artificial neural network [26]. This study also reported that the kindergarten BMI Z-score is more important for BMI prediction than the demographic and socioeconomic variables. Fu et al. developed an ML-based framework to predict childhood obesity by using health examination, lifestyle and dietary habits, and anthropometric measurement-related data [27]. Among the 25 features used in this study, the BMI Z-score trajectory on the first year of life and maternal BMI at enrollment were deemed to be the most important ones. Pang et al. proposed ML models to predict childhood obesity from EHR data [28]. In that study, the authors incorporated seven ML techniques, including XGboost, and the accuracy achieved was 66.14%. Hammond et al. used unaugmented EHR data from the first two years to predict obesity at age five [29]. In this study, different models were developed for boys and girls, and 82% and 76% accuracies were achieved for girls and boys, respectively, by using Lasso regression. LeCroy et al. presented a paper to identify key determinants of childhood obesity using ML [30]. In that study, a simple regression analysis was presented with a low R^2^ value of 0.56.

Machine learning algorithms can be categorized based on their methodology. For example, decision tree, gradient boosting, random forest, and adaptive boosting are commonly known as tree-based ML algorithms. Similarly, artificial neural networks, probabilistic neural networks, and convolutional neural networks are neural network-based ML algorithms. The six most popular types of classification algorithms are based on decision trees, neural networks, probability of occurrence, hyperplanes, neighborhood-based nonparametric approaches, and unsupervised learning. In this study, the goal is to use at least one ML algorithm from each of the six categories. We choose random forest, artificial neural networks, logistic regression, support vector machine, *k*-nearest neighbors, and *k*-means clustering because they are the most commonly used ML algorithms in the decision tree, neural network, probability of occurrence, hyperplane, neighborhood, and unsupervised learning based categories, respectively. These algorithms have been widely used in previous studies for predicting obesity [20,22,23,25,26,31]. Although these six ML algorithms have been used in multiple studies, the implementation approaches differ from one study to another. The features used in these previous studies are also different. In this study, we evaluate how well our implementations of these six ML algorithms predict childhood obesity with available data in multiple possible application scenarios, i.e., data availability for only single, early (prior to two years of age), or multiple random well-child visits. We compare the performances of the ML algorithms and propose the best performing model for each of the application scenarios. Here, the term “ML models” is used to denote the hyperparameter-tuned ML algorithms appropriate for specific application scenarios.

Predicting early childhood BMI can be difficult because the measure of length/height varies dependent upon child positioning; there is also disagreement about norms between genders, and adiposity rebound post-birth creates complexity in determining appropriate growth. Predicting childhood obesity becomes much tougher if sufficient longitudinal growth data and demographic, socioeconomic, and parents’ health and education-related information are not available. Previous studies attempted to predict childhood obesity with all or most of the well-child visit data up to a certain age [32]. Previous studies also required information on a large number of variables related to a child’s growth, socioeconomic conditions, and other family-related factors. We have developed ML models that predict childhood obesity at age five with high accuracy by using single, early, and multiple random well-child visits’ data. The developed ML models use limited information such as birth height, weight and BMI, gestational age, BMI on different DSBs, and gender. After an extensive review of the related literature, we found that this flexible approach of providing prediction models for different application scenarios was not adopted in previous studies and this implementation approach is unique to our study. To the best of our knowledge, this study is the first to predict childhood obesity with only single well-child visit data and some random well-child visit data.

## 3. Methodology

### 3.1. Data Acquisition

**Figure 1:**
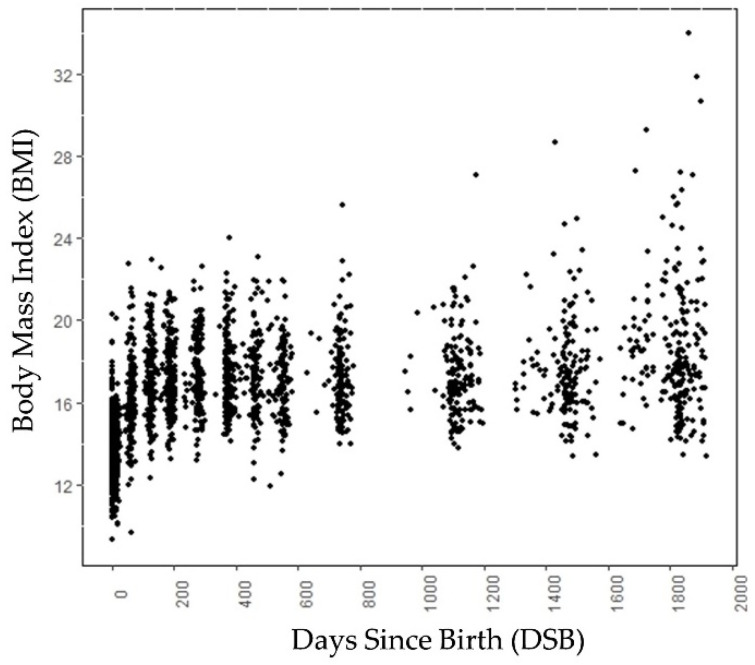
Scatter plot of data samples (BMI) of children at different DSB. Note that the data points are not at exact intervals, i.e., each subject has data points for different DSB.

A longitudinal retrospective chart review of well-child visits to a Midwestern health maintenance organization from birth to 5 years was conducted. Approval was received from the health maintenance organization’s full Institutional Review Board. Data used for this study were deidentified. All records of mothers and children born between 1997 and 2001, an era of increasing childhood obesity, were reviewed. The data contained weight and height information on different days since birth (DSBs) collected during the well-child visits, ranging from birth to around age 5, for 224 children. For each child, we had data on 9 different DSBs on average. For a child, the BMI at around 730 DSB was a child’s two year BMI and the BMI around 1825 DSB was the BMI at five years of age. The birth height, birth weight, gender, and gestational age were also known for these children. Among these 224 children, 110 children were male and 114 children were female. A total of 61 children had normal weight, 47 children were overweight, and the remaining 116 children were obese at age 5. Figure 1 shows the BMI values at different DSBs (days since birth) for these 224 children. The labeling of weight categories at age 5 is based on CDC guidelines (found on the CDC webpage https://www.cdc.gov/growthcharts/clinical_charts.htm, accessed on 30 June 2022) and was performed by experts in the field.

**Figure 2:**
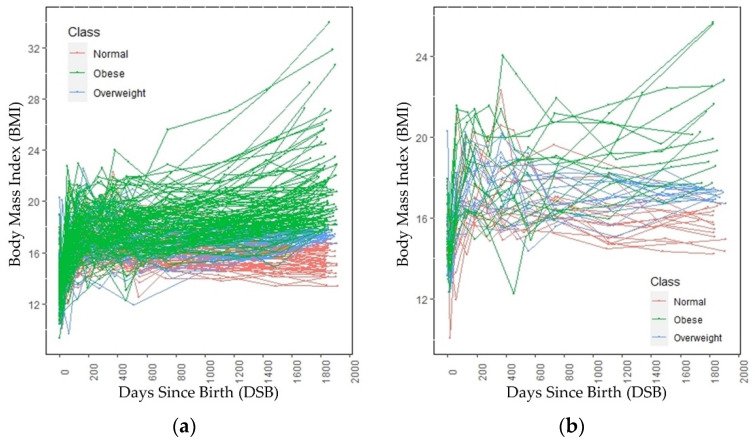
BMI trends through age 5 across (**a**) all subjects and (**b**) a randomly selected subset of subjects.

The growth trends of these 224 children are shown in Figure 2a. Figure 2b shows the growth trend lines of 15 children from each category, selected randomly. Figure 2b is added to show the growth trend lines of all the categories, because the growth trend lines of children in the overweight category overlap the growth trend lines of children in the normal and obese categories in Figure 2a. Both in Figure 1 and Figure 2, 0 DSB means the child’s day of birth. The obesity categories of these children refer to the category to which they belong at age 5, not the category to which they belong at a particular DSB. Up to 700 DSB, it is often very difficult to separate the growth trend lines of the children because they often overlap. After 700 DSB, the distinctions between the normal weight and obese categories are clearly visible.

### 3.2. Data Interpolation and Augmentation

Due to the limited number of data points, we incorporated a data interpolation technique that preserved the data distribution observed in the original data in order to augment the data. Data augmentation reduced the data imbalance and increased the generalization of data.

**Figure 3:**
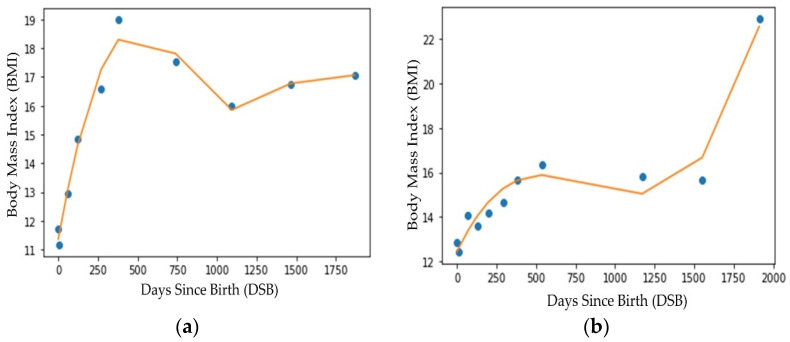
Regression line fitting (3rd degree polynomial) for two different subjects (**a**) child 1 and (**b**) child 2.

To interpolate missing data points, i.e., missing well-child visits, we created a 3rd degree polynomial curve for each child using the BMI values collected during the well-child visits. Figure 3 shows the polynomial curves for two different children. Note that in Figure 3, the dots represent the actual BMI values on different DSBs (data from well-child visits to a doctor’s office) for two different children. The lines are the 3rd degree polynomial line created using the actual BMI values collected during the well-child visits represented by the dots. The 3rd degree polynomial was observed to provide a better approximation (higher R^2^ value) compared with other interpolation techniques (e.g., 5th degree or logarithmic interpolation). Figure 3a,b show that well-child visits were not made on the same DSBs. Additionally, for both children, there were no data for the 2.5 year’s (approximately 913 DSB) well-child visit. Because either all the well-child visits were not made or the visits were not evenly spaced over time, a polynomial regression line was fitted through each child’s data. From the curve, BMI values at evenly distributed DSBs (common DSBs across all the subjects) were collected [33]. For the ML models used in the early and multiple random well-child visit-based application scenarios, the DSB was considered to be a feature of the data and the BMI values on different DSBs were interpolated from the polynomial lines.

The other issue was that the data set was unbalanced, because, among the 224 children, there were 61, 47, and 116 children, respectively, in the normal, overweight, and obese categories. Different approaches have been taken in previous studies to augment longitudinal data, including undersampling and oversampling [34], mean substitution [35], random effect models [36], and random transformation techniques [37]. We did not choose undersampling, because our dataset was relatively small and this would have made it even smaller. Since we only had a limited number of subjects in each class, oversampling from these small sets would have reduced intraclass variability [34]. In mean substitution, the missing observations of a particular variable are replaced by the average of the observed data of that variable in the other subjects. In this paper, the goal was to create new subjects, so there was an equal number of children in the normal weight, overweight, and obese categories. Using the mean substitution method, creating multiple subjects was impossible as there was only one mean value for a particular DSB. By using the mean substitution method, only one subject could be created in each category. The random effect model assumes that the response variable has a fixed relationship with the explanatory variable across all observations, but these fixed effects may vary from one observation to another observation. We did not choose this approach, because not only were the variance components different from one BMI interval to another but also there were significant differences between the mean values on different DSB intervals. For example, the BMI distribution between 1 and 100 DSB was different from the BMI distribution between 701 and 800 DSB. Our intention was to choose an approach that allowed considering different distributions on different longitudinal phases (different DSB intervals).

To balance the data among the different categories (in order to have an equal number of subjects from the normal, overweight, and obese categories), we incorporated a data augmentation technique that used ideas based on random transformation methods that preserved the distribution of data for each category on different DSB intervals. In this technique, we fitted a normal distribution to the existing data and then randomly sampled from this normal distribution, truncating the distribution by providing ceiling and floor values that limited the maximum and minimum values for each category, to generate augmented data points. For example, to create data representing a new child who was overweight, we divided the five-year period into smaller intervals of 100 days. Then, for every 100 DSB we fitted a normal distribution curve to the data of the existing children who were overweight. For example, if there were 200 BMI data points for children who were overweight from 1 to 100 DSB, then these 200 data points were fitted to a normal distribution for that interval. To avoid problems with outliers, we imposed upper and lower limits on the values sampled from the normal distribution, effectively making it a truncated normal distribution. The same technique was used to obtain BMI values for all of the intervals, resulting in data for a new child who was overweight based on the existing overweight category data. The main advantages of this augmentation approach were that it created balanced datasets across different categories without outliers and preserved the data distribution characteristics on different DSB intervals.

For the interpolated data used in application scenarios 2 and 3, we did not include other features, e.g., birth height, birth weight, gestational age, or gender. For these two application scenarios, we only considered the longitudinal characteristics of BMI and DSB data using the BMI values at different DSBs as feature values. Sometimes, additional information other than BMI may not be available; the models used in application scenarios 2 and 3 could predict obesity categories effectively in such situations, simply because they were trained and evaluated to make predictions using solely BMI information.

### 3.3. Model Validation

For determining the obesity category from a single well-child visit, early well-child visits, and multiple random well-child visits, a 70%–30% train test splitting was used, where the data were split randomly into 70% training data and 30% test data.

### 3.4. Application Scenarios

In this paper, we proposed different ML models to classify the subjects into obesity categories for the following 3 application scenarios.

1. Determining the obesity category from a single well-child visit.
2. Determining the obesity category based on multiple early well-child visits.
3. Determining the obesity category based on multiple random well-child visits.

#### 3.4.1. Determining the Obesity Category from a Single Well-Child Visit

In this application scenario, we focused on determining the obesity of a child at age 5 based on the data from a single well-child visit. This approach is important to consider, because, many times, information on multiple well-child visits may not be available and only the current visit data and birth data may be known. Therefore, our first approach was to determine the obesity category trend of the child at age 5 based on the available data from the current well-child visit. We included not only the BMI and respective DSB but also other features, e.g., gestational age, birth BMI, birth height, birth weight, and gender. Six different classification algorithms—logistic regression, support vector machine (SVM), random forest, artificial neural network (ANN), *k*-means clustering, and *k*-nearest neighbors—were used to predict the obesity categories at age 5. All these algorithms were developed in a way that they predicted categories by using the current DSB and BMI, birth weight and height, gestational age, and gender as features.

The implementations of the aforementioned ML algorithms were performed using Python (version 3.0) with the Scikit learn platform (Version 1.1.2) [38]. Initially, default hyperparameters were used for these classification algorithms, as discussed later. These algorithms were not given any information regarding which data were from which children. For example, if there were data on 10 different DSBs for a child, the algorithms did not know that these data were from the same child. That means these algorithms considered that each subject had one DSB, one BMI, gender, gestational age, and birth weight and height. Combining all the 224 children, we had BMI data on 2039 DSBs. The data were fed into the ML models in a way that the algorithms considered 2039 subjects. DSBs from the same child had the same birth and gender information. For example, if among the 2039 subjects, the first 9 were from the same child, the birth and gender information was the same for these 9 subjects. The overall accuracy, category-wise precision, recall, and F1 scores were compared among these algorithms and the best performing algorithm was proposed. After that, how the proposed algorithm performed on different ages was analyzed. Some of the hyperparameters of the proposed algorithm were tuned to maximize the predictive ability of the models. The performance of the proposed algorithm was also analyzed with different random seeds to ensure that the proposed algorithm performed consistently.

#### 3.4.2. Determining Obesity Category Based on Early Well-Child Visits

In this application scenario, we presented a unique ML algorithm for the detection of childhood obesity at age five, based on the BMIs (collected during the well-child visits) up to two years of age. Observing the raw data trend of the children in Figure 2, it was evident that there was no clear trend in the first few DSB points, especially up to two years of age. However, as DSB increased, the differences between the trend lines of different categories (normal, overweight, and obese) became visible. Therefore, it was possible to predict the obesity category of a child based on only a few data points, but predicting the category accurately using only BMI values prior to 2 years of age was difficult because of the overlapping growth lines. In our proposed method, we evaluated interpolated (data points calculated from a polynomial regression line) and both balanced (equal number of subjects from each category) and unbalanced (unequal number of subjects from each category) data for up to two years of age to predict the obesity category at age 5. DSBs prior to two years of age were considered as features and respective BMI values were the feature values. Birth BMI was also added to the model as the BMI on 0 DSB. Six different classification algorithms—logistic regression, artificial neural network, support vector machine, *k*-nearest neighbor, *k*-means clustering, and random forest—were used to predict the categories at age 5, using the BMI values up to two years of age.

#### 3.4.3. Determining Obesity Category Based on Multiple Random Well-Child Visits

According to the American Academy of Pediatrics (AAP), well-child visits up to five years of age should be scheduled for the first week, 1 month, 2 months, 4 months, 6 months, 9 months, 12 months, 15 months, 18 months, 2 years, 2.5 years, 3 years, 4 years, and 5 years of age [39]. Nonetheless, not all children may have well-child visits at the prescribed intervals, rather they may have multiple well-child visits at random intervals. It is difficult for any model to predict the obesity category accurately based on random visit data. However, we formulated a model that utilized the data from the random DSB intervals from all the subjects to obtain trends. At first, this model used a third-degree polynomial equation to create a fitting line for each child. Then, the algorithm collected BMI values for specific DSBs from the fitting lines of the children. Basically, the algorithm made a classification based on the projected data collected from the fitting lines of an individual child. This technique enabled the model to predict the weight category accurately. In our proposed method, we evaluated interpolated and both balanced (equal number of subjects from each category) and unbalanced (unequal number of subjects from each category) data to predict the obesity category at age 5. Artificial neural network, *k*-nearest neighbor, *k*-means clustering, random forest, support vector machine, and logistic regression were used to predict obesity at age 5. Finally, the hyperparameter-tuned version of the best performing model was used to predict obesity at age 5. For predicting the obesity category, DSBs were considered as features. BMI values at the DSBs used as features were the feature values. Birth BMI was also added to the model as the BMI for 0 DSB.

We performed feature scaling, because ML algorithms converge faster when the feature values are on a relatively similar scale [40]. Here, the feature value for a subject was standardized by subtracting the mean and dividing it by the standard deviation, meaning the classification algorithms for all three application scenarios (single well-child visit, early well-child visits, and multiple random well-child visits) made predictions using standardized feature values.

Table 1 shows what type of dataset was used in the different application scenarios. A balanced dataset was not used on a single well-child visit-based application scenario because our approach to data balancing was not suitable for creating subjects with a single well-child visit. In addition, for a single well-child visit-based application scenario, we had a relatively good number of subjects from all three categories. The dataset size for a single well-child visit was significantly larger than the early-well child and multiple random well-child visit-based application scenarios. There were more well-child visit data per subject for the multiple random-well child visits’ application scenario than the early well-child visits’ application scenario, because, in the early well-child visit-based application scenario, well-child visits after two years of age were not included. The balanced data had 450 subjects and each category had 150 subjects. The input features used in different application scenarios are also shown in Table 1.

### 3.5. Classification Algorithms

#### 3.5.1. Random Forest (RF)

Random forest [41] is an ML algorithm that consists of a large number of individual decision trees created by using random subsets of data, commonly known as bagging, that operate as an ensemble. Random forest is an extension of bagging as it builds trees by not only taking random subsets of data but also by taking random selections of features. Each individual tree in the random forest delivers a category prediction and the category with the most votes becomes the model’s prediction. This approach of aggregating the results collected from all the trees is very effective in reducing variance. Previous studies have observed that random forest is robust in dealing with sparse and noisy data and is resilient against outliers [42].

**Figure 4:**
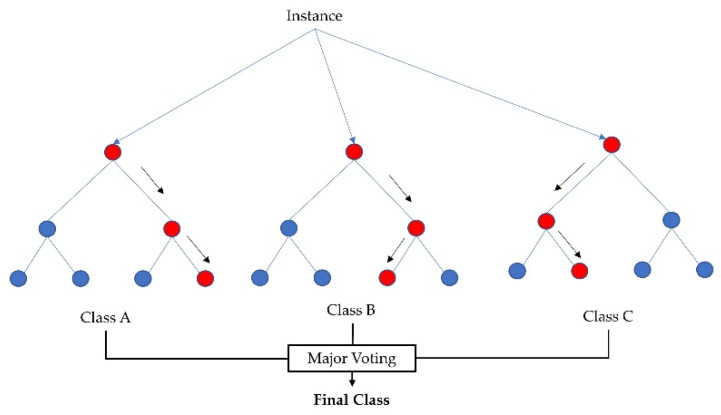
Random forest classifier using several decision trees and major voting for accurate classification.

As random forest selects features randomly from a handful of features, it is imperative to obtain the feature pool based on importance. The random forest implementation used the Gini index [43], i.e., the features were taken at each node, which resulted in the lowest impurity (Gini index). Therefore, from the total number of features we acquired features randomly for the feature pool, calculated the accuracy of randomly taken features, and went back to select those features which yielded better accuracy. Figure 4 shows an example of a random forest model that fits a number of decision tree classifiers on various subsamples of the dataset (instances) and uses major voting to improve the predictive accuracy and to control overfitting.

#### 3.5.2. Logistic Regression (LR)

**Figure 5:**
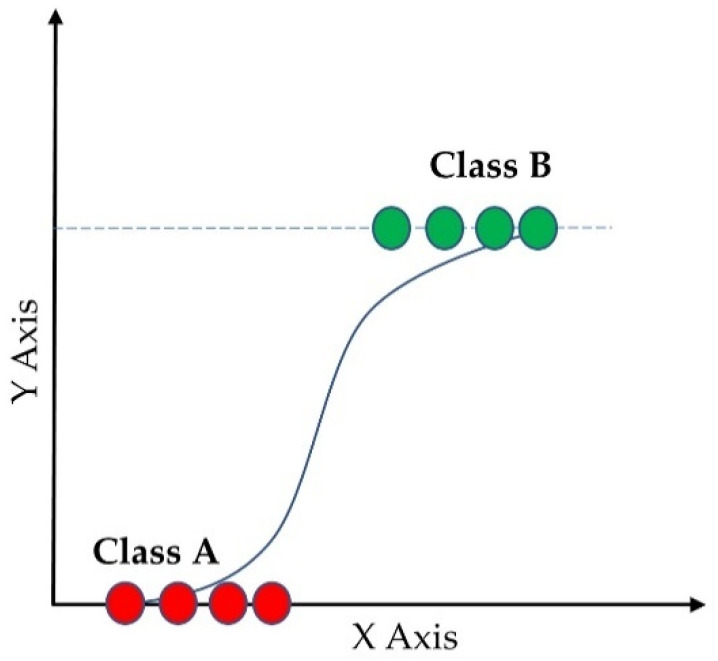
Logistic regression of binary classification.

Logistic regression [44] is one of the most common supervised ML classifiers that estimates the probability of an event occurring (in our case, a prediction of category of a subject into normal, overweight, and obese) based on a given dataset of independent variables (the features mentioned earlier). Figure 5 shows the probability of the discrete outcomes, marked by red circles, given the input variables for a binary classification problem. Here, the green circles are classified in one class and the red circles are classified in the other class. Since we had three categories, multinomial logistic regression was used. The sigmoid function showed in Equation (1) was used as the logistic function.

(1) $$\sigma \left(t\right)=\frac{1}{1+e^{-t}},$$

where *t* is the linear combination of multiple independent explanatory variables.

#### 3.5.3. Support Vector Machine (SVM)

**Figure 6:**
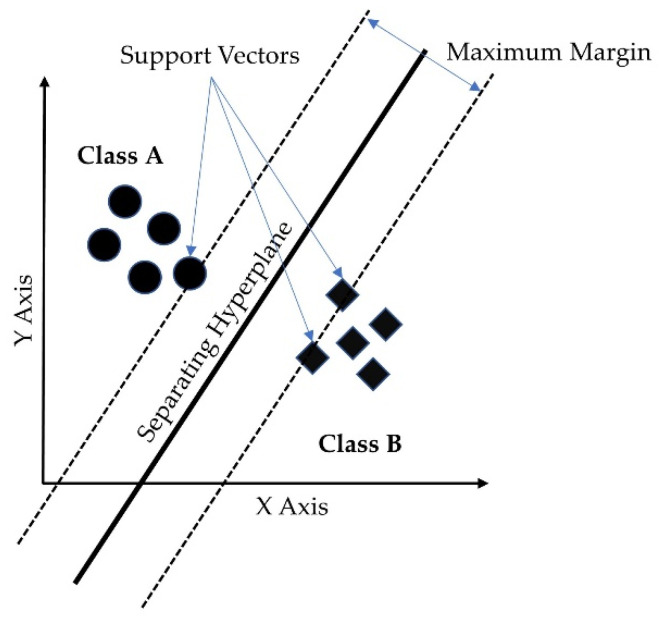
Support vector machine hyperplane for binary classification.

The support vector machine (SVM) is a supervised learning model with associated learning algorithms that analyzes data for classification and regression analysis [45]. A support-vector machine maps data to a high dimensional feature space so that data points of different categories can be separated. The separator in the SVM is a hyperplane, thus, the objective of the support vector machine algorithm is to find a hyperplane in an N-dimensional space (N being the number of features) that distinctly classifies the data points. Figure 6 shows the separating hyperplane derived from the maximum margin separator and the support vectors for a 2-class classification problem. SVM is a binary classification algorithm, but it can handle multicategory problems. In multicategory classification, SVM breaks multicategory problems into several binary classification problems [46]. There are two ways to handle multicategory problems in SVM. One approach is to create binary classification problems for each pair of categories, commonly known as a one-vs-one approach. In the second approach, the multicategory problem is transformed into a binary classification problem for each category meaning the outcome is this particular category or not, this approach is commonly known as one-vs-rest. The second approach was used in our model. A radial basis function (RBF) kernel was used in this study.

#### 3.5.4. Artificial Neural Network (ANN)

**Figure 7:**
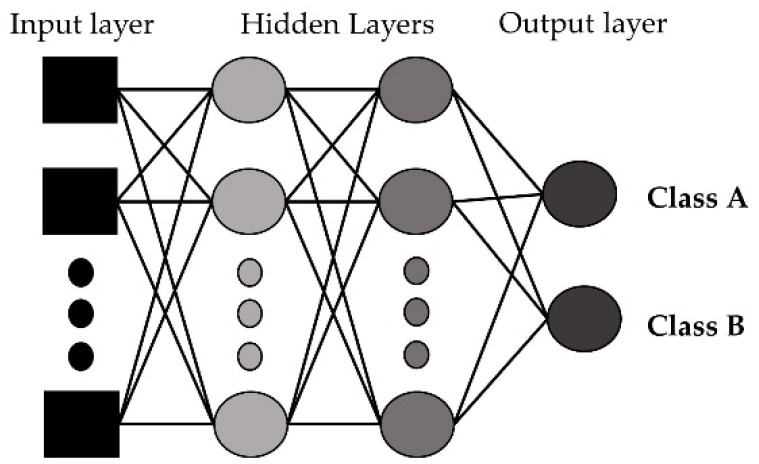
Artificial neural network with input, hidden, and output layers.

An artificial neural network imitates how the nerve cells function in a human brain [47]. An ANN is a network of interconnected neurons and the neurons are connected to one another. In an ANN, there are input, hidden, and output layers. The input layer is the initial data, the hidden layer(s) performs necessary computations, and the output layer generates the results for the given inputs. In our developed model, we used 3 hidden layers as a multilayer perceptron and a sigmoid function for activation. Figure 7 shows the basic structure of the artificial neural network with the input layer, hidden layers, and output layers for a binary classification problem.

#### 3.5.5. *k*-Nearest Neighbors (*k*-NN)

**Figure 8:**
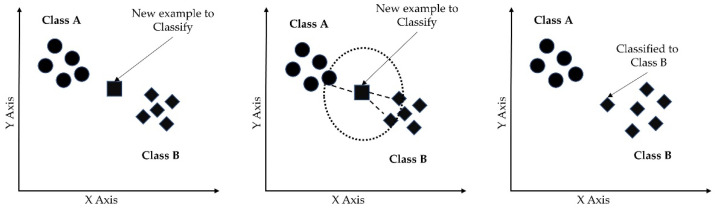
*k*-nearest neighbor algorithm for binary classification.

The *k*-nearest neighbor algorithm (*k*-NN) is a non-parametric supervised learning method used for classification. The *k*-nearest neighbor (*k*-NN) algorithm creates a boundary to classify the data based on nearest neighbors [48]. When new data points come in, the algorithm assigns the new data to the category that is most common among its *k* nearest neighbors. Prediction is based on the nearest *k* neighbors calculated by the hyperparameter *k*. Larger *k* values result in smother curves of separation, resulting in less complex models. Figure 8 shows the basic functioning of the *k*-NN algorithm. The class of an unknown instance (marked by the square) is determined by the class of *k* of its nearest neighbors.

#### 3.5.6. *k*-Means Clustering

**Figure 9:**
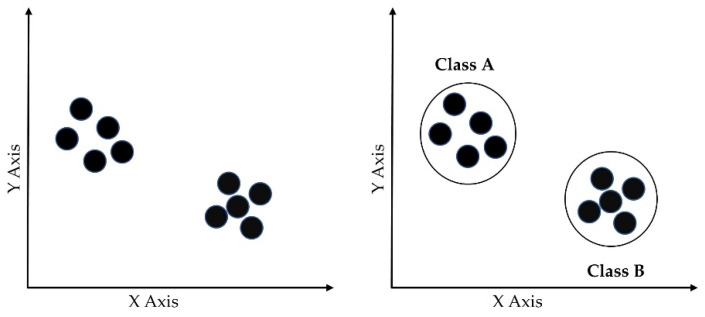
*k*-means clustering algorithm for a 2-class problem.

*k*-means clustering is an unsupervised ML algorithm that tries to partition *n* observations into *k* clusters, in which each observation belongs to the cluster with the nearest mean (cluster center or cluster centroid). In other words, observations are assigned to the clusters in a way that minimizes the sum of the distances between the observations and the centroids of the clusters to which they belong. Figure 9 shows the clusters for a 2-class classification problem using the *k*-means clustering algorithm. Since there are two classes, there are two centroids, and clusters were generated depending upon the distances from the centroids.

### 3.6. Hyperparameter Selection

For preliminary model assessment, the models were evaluated using their default hyperparameter settings. After the performance evaluation, the best performing model was selected for further tuning of the hyperparameters. Finally, the results were presented for the model that had tuned hyperparameters.

## 4. Results and Discussion

The following performance metrics were used to analyze how well the ML algorithms classified children into normal weight, overweight, and obese categories.

**Accuracy:** Accuracy determined what fraction of the samples was predicted correctly [49]. Equation (2) shows the function used to calculate accuracy. A *TP* (true positive) occurred when the model correctly predicted the positive categories (for example predicted obesity at age 5 when the child was actually obese at age 5), whereas a *FP* (false positive) occurred when the model incorrectly predicted the positive categories. Similarly, a *TN* (true negative) occurred when the model correctly predicted the negative categories, whereas a *FN* (false negative) occurred when the model incorrectly predicted the negative categories.

(2) $$Accuracy=\frac{\left(TP+TN\right)}{\left(TP+\mathrm{TN}+FP+FN\right)}$$

**Precision:** Precision is the ability of a classifier not to label an instance positive that is actually negative. It is the accuracy of the positive predictions [49]. Equation (3) shows the function used to calculate precision.

(3) $$Precision=\frac{TP}{\left(TP+FP\right)}$$

**Recall:** Recall is the ability of a classifier to find all positive instances [49]. It is the fraction of positives that are correctly identified. Equation (4) shows the function used to calculate recall.

(4) $$Recall=\frac{TP}{\left(TP+FN\right)}$$

**F1 Score:** The F1 score is a weighted harmonic mean of precision and recall [49]. It is mainly used to compare the performance of two different classifiers. One classifier may have better precision and another one may have better recall. In such cases, the F1 score can be used to find out which classifier performs better. Equation (5) shows the function used to calculate the F1 score.

(5) $$F1\;Score=\frac{2*\left(Recall*Precision\right)}{\left(Recall+Precision\right)}$$

### 4.1. Determining the Obesity Category Based on a Single Well-Child Visit

Classification accuracy and category-wise precision, recall, and F1 scores for the different ML algorithms are shown in Table 2. *k*-means clustering had the lowest accuracy. Support vector machine and *k*-NN performed better than logistic regression and ANN, but still were restricted to less than 70% accuracy. Random forest performed well compared with the other classification algorithms. Therefore, random forest is proposed as the classification algorithm for single well-child visit-based prediction.

For a single well-child visit, random forest correctly predicted 89% of the time. One constant characteristic across these algorithms was the lower recall values of the overweight category. Logistic regression corresponded to a recall value of only 4% for the overweight category, which means that only 4% of the children who were overweight at age five were predicted correctly and 96% of children who were overweight were either predicted as normal weight or obese. Due to the nature of the given data, logistic regression could not differentiate the overweight category from the normal and obese categories and, therefore, assigned a very low probability to the overweight category. Random forest had very high recall values for the normal weight and obese categories but relatively lower recall values for the overweight category. This is because the growth trend lines of children who were overweight at age five, as shown in Figure 2, overlapped with children in the normal weight and obese categories. However, there were clearer distinctions between the growth trend lines of the normal weight and the obese categories. In addition, the data were imbalanced as there were fewer children in the overweight category than there were children in the normal or obese categories. For these reasons, the algorithms had many false negatives for the overweight category. Random forest had more than 90% precision for the normal and overweight categories but only 84% for the obese category. This means that, of the children who were classified as obese, 84% of them were obese and the other 16% were non-obese. Since 24% of children who were overweight were predicted incorrectly and the growth trend lines overlapped, one can conclude that the random forest algorithm classified most of these children who were overweight as obese. 

**Figure 10:**
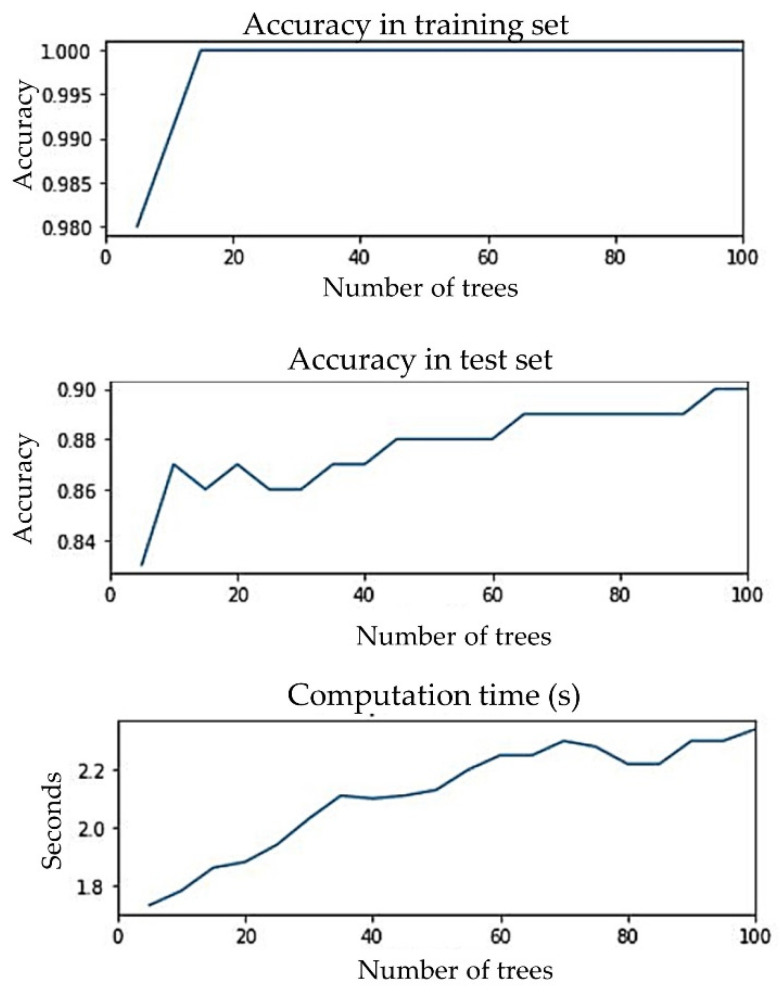
Accuracies of training and test set and computation time for different numbers of trees.

Since random forest was proposed as the classification algorithm for single visit-based prediction, it was important to ensure that the proposed algorithm did not overfit. A suitable number of trees were selected by analyzing the differences between the accuracies of the training and test sets and the computation time for the different numbers of trees, as shown in Figure 10. The number of trees was varied, from 5 to 100. Computation time for 5 trees was 1.73 s and for 100 trees was 2.34 s, meaning the computation time did not increase significantly. The gap between training and test set accuracy was never more than 15%. Training accuracy was about 98% when the number of trees was 5; for 10 or more trees, it was about 100%. Test accuracy was 83% when the number of trees was 5 after that it gradually increased to 90%. We proposed to have 65 trees in the forest, because the test accuracy did not improve much beyond that and the gap between the training and test accuracy was only 11%. We recommend not to set a limit to the depth of the trees. Since computation time was not high, making the ensemble converge a little earlier will not add great value. So, we recommend letting the nodes expand until all leaves are pure or all leaves contain less than the minimum number of samples required to split an internal node.

**Figure 11:**
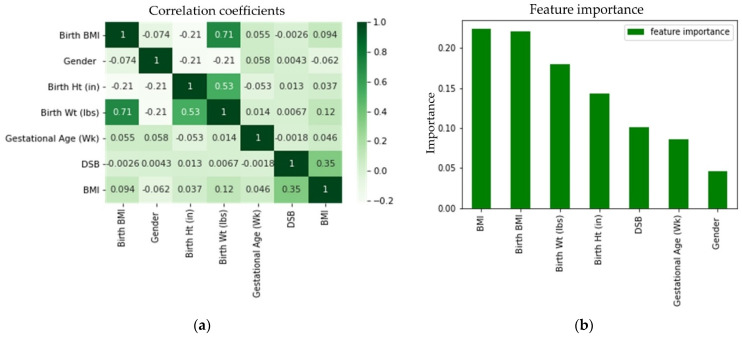
Feature (**a**) correlation coefficient and (**b**) importance for the random forest classifier.

The correlation matrix of the features shown in Figure 11a demonstrated that there was no extremely strong correlation between any two features, although birth BMI was highly correlated with birth weight and birth height was moderately correlated with birth weight. These correlations were expected, because birth BMI was calculated from birth height and birth weight. From Figure 11b, it can be seen that birth BMI, birth weight, and birth height had the second, third, and fourth highest contributions, respectively, to the predictions. In addition to taking a random subset of data, random forest takes a random selection of features rather than taking all features to grow trees. From the seven features that we had in our data, random forest took three (conventionally taken as the square root of the total number of features) random features to grow each tree. Thus, for some trees, birth BMI may not be selected in the same feature pool with the birth weight and, similarly, birth height and birth weight may not be selected together. For those trees where birth BMI and birth weight and birth height and birth weight were not selected together, the predictions were not affected by these correlations. Each tree in the random forest delivered a category prediction and the model’s prediction was made based on the highest votes. Therefore, even though birth BMI, birth height, and birth weight were correlated, all of them contributed significantly to the prediction. Thus, all three of them (birth BMI, birth weight, and birth height) were added in the model. The feature importance based on the impurity measure of the random forest algorithm was evaluated to visualize the importance of the features for classification (see Figure 11b). From Figure 11b, it can be seen that the most important feature for obesity classification was BMI, as anticipated. Furthermore, no single feature acted independently to classify the obesity category for each subject. It was found that gender had the least importance on the prediction. It was observed that the prediction accuracies by gender determined by using separate data were very close and these prediction accuracies were also very close to the combined accuracy. Therefore, it was not surprising that gender had the least importance on the prediction.

Table 3 shows the confusion matrix of the proposed hyperparameter-tuned random forest model. The overall accuracy was 89%. The confusion matrix shows that, among the 35 children in the overweight category who were classified incorrectly, 32 were classified as obese.

The test set was divided into 13 different age groups; the performance of the random forest in the different age groups is shown in Table 4. Except for the first month, the random forest had more than 80% accuracy for all the age groups. From Figure 2, it can be seen that the growth trend lines started around the same place and they overlapped significantly for the early DSB values, which is why random forest had relatively low accuracy in the first month. On average, predictions were better as DSB increased, although there were some exceptions.

The proposed random forest algorithm was run with 10 different random seeds to check the consistency of its performance; the results are shown in Table 5. The average accuracy was 91% and the average precision and recall values were more than 80%. Among the 10 replications, the minimum accuracy was 87%. Among the 10 replications, the minimum precision and recall values were more than 80%, except for the recall value of the overweight category. The minimum recall value of the overweight category among these 10 replications was 76%. Among the average precision values, the average precision of the obese category was the lowest. Among the average recall values, the average recall of the overweight category was the lowest. These characteristics were consistent with the confusion matrix shown in Table 3. So, it can be concluded that the proposed random forest algorithm performed well consistently.

### 4.2. Determining the Obesity Category Based on Early Well-Child Visits

We evaluated all six models on unbalanced and balanced datasets and the performance evaluations are shown in Table 6 and Table 7, respectively. For both of the datasets, random forest performed the best. Random forest exhibited better accuracy with the balanced data compared with the unbalanced data.

**Figure 12:**
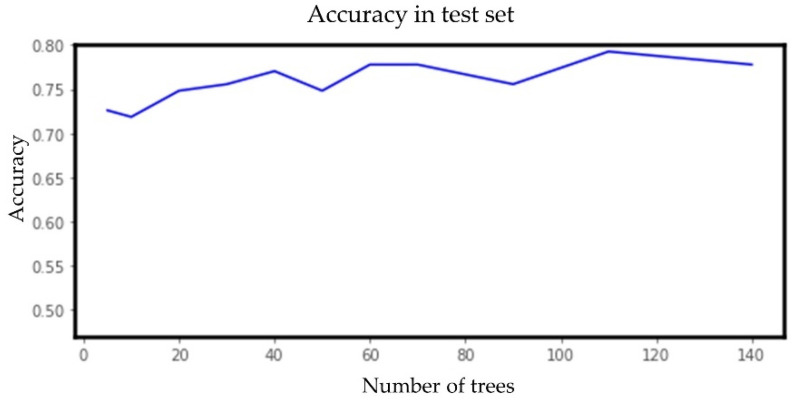
Accuracy vs. number of estimators (trees) for the random forest model for the early well-child visit scenario.

The LR, SVM, RF, ANN, *k*-NN, and *k*-means clustering algorithms were evaluated using their default hyperparameter setting. For example, the number of hidden layers for ANN was taken as (30, 30, 20) and, for the logistic function, a sigmoid function was taken. For the implementation of a support vector machine (SVM), a RBF kernel with hyperparameters C as 1 and gamma as scaled 0.1 was chosen for its adaptability on stochastic data [50]. For *k*-NN, the neighbor size was selected to be 30. For *k*-means clustering, the cluster number was set to 3. Since random forest performed the best among all the six classifiers and also performed better with balanced data compared with unbalanced data, we tuned the hyperparameter (number of estimators) of random forest for the balanced dataset. Figure 12 below shows the change in accuracy with varying numbers of estimators. We selected the number of estimators to be 64, as increasing the number of estimators further only marginally improved accuracy.

**Figure 13:**
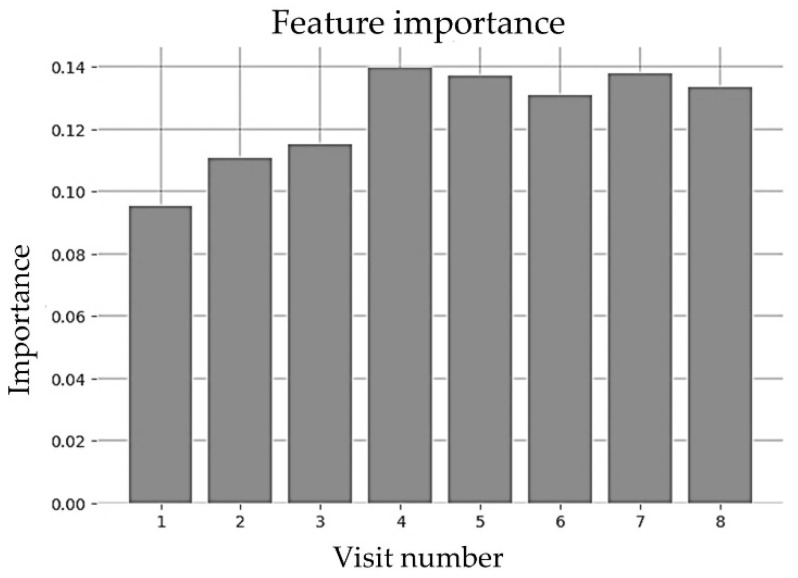
Feature importance of random forest for early visits (until 2 years).

The random forest model implemented in this paper used the Gini importance measure [51], where features were selected based on a mean decrease in impurity or entropy. Moving down for each tree led to decreased impurity or uncertainty that yielded better classification accuracy at each node. The Gini index displayed the probability that a certain feature was incorrectly classified when selected randomly. Looking at the impurity-based feature importance of the random forest implementation shown in Figure 13, it is clear that the BMI collected in a specific well-child visit did not provide significantly more importance than the other visits.

Finally, the hyperparameter-tuned random forest classifier trained on balanced data was proposed for childhood obesity prediction for the early well-child visit scenario. The confusion matrix for testing data with the random forest classifier is shown in Table 8. The overall accuracy was 77%. Predicting the obesity category accurately with early data points was very difficult. Figure 2 shows that, in the early years, the growth trend lines of normal, overweight, and obese categories overlapped. In fact, the trend lines started almost from the same place. In addition, it is clear that, in the early stages, the growth trend lines were turbulent and became smooth at about 2 years of age. Since multiple BMI values prior to two years of age were used and these BMI values fluctuated significantly, the classification accuracy was lower compared with the other two application scenarios.

### 4.3. Determining the Obesity Category Based on Multiple Random Well-Child Visits

The prediction models’ performance on unbalanced and balanced data is shown in Table 9 and Table 10, respectively. Assessing the evaluation parameters, it was observed that random forest provided the best metrics for childhood obesity category prediction based on multiple random visits. Random forest had better accuracy with the unbalanced data compared with the balanced data. Our evaluation indicated that *k*-means clustering performed poorly in contrast to *k*-NN. This is because *k*-NN relied on the similarity of the BMI values of a category at a certain DSB evaluated from the neighboring data points, whereas *k*-means clustering focused on the mean distance of the BMI from the cluster mean of a category. The proximity of a BMI value to the neighboring members of a category at a specific DSB was a more dominant factor to predict the obesity category than the distance to the cluster center, which provided better results for *k*-NN.

**Figure 14:**
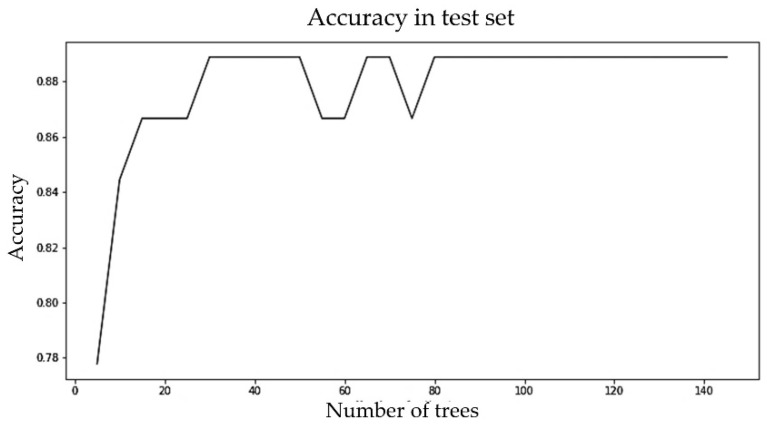
Accuracy vs. number of estimators (trees) for the random forest model for the multiple random well-child visit scenario.

The ML algorithms were evaluated using the default hyperparameter settings mentioned earlier. Since random forest performed best among all the six classifiers and also performed better with unbalanced data compared with the balanced data, we further tuned the hyperparameter (number of estimators) of random forest for the unbalanced dataset. We selected the number of estimators to be 30, as increasing the number of estimators further did not increase the accuracy. BMI values on nine randomly chosen different DSBs ranging from 0 to 1650 were used to predict the obesity category. Figure 14 shows the change in accuracy with a varying number of estimators.

**Figure 15:**
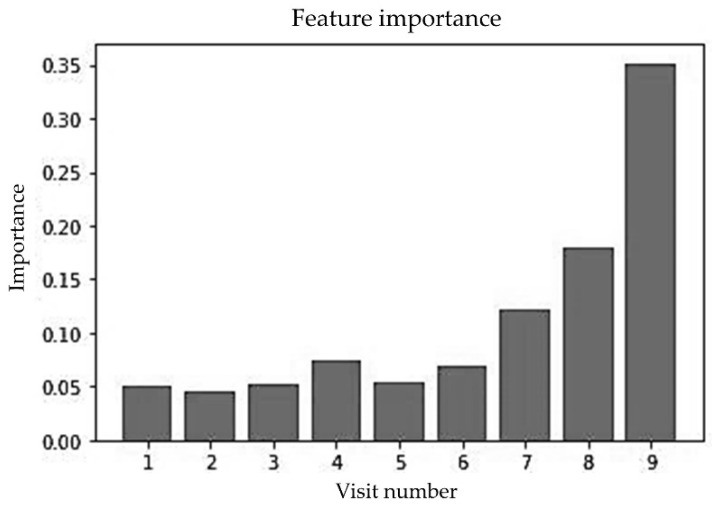
Feature importance of random forest for multiple random visits (unbalanced data).

Figure 15 shows the impact of well-child visits that were conducted between birth to 1650 DSBs on prediction. The first six visits conducted within the first 2.5 years of a child’s life had lower importance than later visits. One thing to notice is that the first six visits had almost the same level of importance and after that the importance increased as the visit number increased. The latest visit had the highest importance. This is not surprising, because the latest visit was conducted at an age that was closer to 5 years of age than the earlier visits.

Finally, the hyperparameter-tuned random forest classifier trained on unbalanced data was proposed for childhood obesity prediction for the multiple random well-child visit scenario. Note that other features (e.g., gender or gestational age) were not used, thus the random forest implementation relied solely upon the BMIs at different DSBs. Table 11 shows the performance of the parameter-tuned random forest model for the unbalanced dataset. The overall accuracy of the random forest model was 89%.

### 4.4. Proposed Models for Different Application Scenarios

In the data, there were frequent well-child visits up to two years of age and between two to five years there were very few (three or four) well-child visits. For example, there were 947 and 434 BMI data points between 1 and 365 DSB and 366 and 730 DSB, respectively, whereas there were 149, 171, and 206 BMI data points between 730 and 1095, 1096 and 1460, and 1461 and 1825 DSB, respectively. Thus, there were less data after two years of age. The augmentation process used in this study could not regenerate BMI data effectively after two years of age due to not having enough data points. The normal distribution curves found in the early intervals were better at capturing the nature of the data than the normal distribution curves found in the later intervals because there were more data to use in fitting the normal distributions. Therefore, the balanced data after two years of age were not as good as the data prior to two years of age. This is the reason why the prediction results were better for early child visits compared with multiple random visits with the balanced data.

The random forest algorithm was implemented on both balanced and unbalanced data for the last two application scenarios: early well-child visits and multiple random well-child visits. Random forest was chosen because of its better performance [52] in handling data with high variance and uncertainty. Using the bagging technique results in accurate and stable predictions. For the application scenario of childhood obesity prediction using early well-child visits, balanced data showed better performance compared with unbalanced data, as the balanced data could capture the data distribution effectively under 2 years of age. Therefore, the balanced data model was chosen and the hyperparameter was tuned for the proposed application. For the application scenario of childhood obesity prediction using multiple random well-child visits, an unbalanced data model was proposed. Random forest has been observed to perform better on unbalanced data rather than balanced data for this dataset due to the challenges in balancing the data from over two years of age and the hyperparameter was tuned for the proposed application scenario. 

## 5. Conclusions

In this paper, we proposed three different ML models for three different application scenarios (prediction with single well-child, early well-child, and multiple random well-child visit data) for assessing obesity category (normal, overweight, and obese) for children at age five. We evaluated multiple ML algorithms for each of these application scenarios and recommended the models that provided the best accuracy. 

Over the years, numerous ML models have been developed to predict childhood obesity. Previous studies were mainly focused on developing ML models that predict childhood obesity at a certain age by using all the well-child visit data up to that age (e.g., predicting the obesity category at age five when a child is age two by using all the well-child visit data up to age two). Most of the previous studies used a wide range of factors such as growth trend, socioeconomic condition, family health issues, and eating patterns. Data on socioeconomic, family health, and eating pattern-related factors might not always be available. In addition, all the well-child visit data may not always be available. This study provides better usability compared with previous studies, because the proposed methods make good predictions using only limited features (e.g., birth weight, height and BMI, gender, gestational age, and BMI) and limited longitudinal data; we even proposed an ML model that makes good predictions with only data from a single well-child visit. 

An exact comparison with other methods was not possible due to our different input datasets. However, the models proposed in this study were evaluated using the same performance parameters (accuracy, precision, recall, F1 score, and confusion matrix) used in the previous studies. Our prediction models for single well-child visits, early well-child visits, and multiple random well-child visits can predict obesity at age five with an accuracy of 89%, 77%, and 89%, respectively. There are four specific reasons why our models achieved good prediction accuracies. First, we developed fine-tuned ML models by ensuring appropriate values of the hyperparameters for the available features, which provided good prediction results without causing overfitting. Second, we only used relevant and high-impact features. We only added those features in the model that helped to improve the accuracy. Third, there was no extremely strong correlation between any two features. Fourth, interpolating BMI values from the third-degree polynomial equation helped to generate interpolated BMI values preserving the original BMI trend for the well child-visits that were not made. This interpolation technique enhanced the prediction quality for the early well-child visit and multiple random well-child visit-based prediction models. In the single well-child visit-based prediction model, birth weight, birth height, birth BMI, gestational age, gender, BMI, and age as day since birth (DSB) were used as features, whereas in the early and multiple random well-child visit-based models only BMI values at different DSBs were used. Overall, it has been observed that random forest outperformed other conventional ML algorithms due to its superior capability to handle uncertain and high variance data. Specifically, random forest builds multiple decision trees and merges them together to provide a more accurate and stable prediction.

There are four major contributions of this study. First, this study proposes three different prediction models for three different application scenarios: predicting childhood obesity using data from a single well-child visit; early well-child visit data, meaning data from well-child visits up to two years of age; multiple random well-child visit data, meaning the data from some of the well-child visits are missing. To the best of our knowledge, this study is the first to predict childhood obesity with only data from a single well-child visit and some random well-child visits. Second, this study provides a basis for how to interpolate missing data points using polynomial regression curves without compromising the characteristics of the original data. Third, this study provides a unique data augmentation approach that can be used to generate new subjects to create a balanced dataset. This study also illustrates how to select a proper data augmentation approach for similar studies. Fourth, unlike previous models, the proposed machine learning models predict childhood obesity using basic information such as height, weight, BMI, gender, and gestational age. This study explains how good prediction accuracy can be achieved by effectively using basic information such as anthropometric measurements, gender, and gestational age.

The main limitation of this study was having data on only 224 children and the categories not being balanced. We intend to strengthen our models by conducting a future analysis using data for significantly more subjects. Recent studies showed, that in some situations, ML ensemble methods that combine multiple ML algorithms might perform better than individual ML algorithms. Ahmed et al. developed seven ensemble models combining different ML algorithms, including naïve Bayes, decision tree, random tree, random forest, support vector classifier, logistic regression, adaptive boosting, gradient boosting, support vector machine with sequential minimal optimization, and JRip, to detect Android ransomware and to mitigate adversarial evasion attacks and found that the ensemble methods performed better than the individual ML algorithms [53]. Djenouri et al. used several deep learning architectures such as VGG16, RESNET, and DenseNet with an efficient ensemble learning and attention mechanism and achieved a disease detection rate of 92% [54]. Once we obtain additional data with significantly more subjects, we will investigate hybrid ensemble methods and check whether they perform better than the ML algorithms proposed in this paper. We also intend to develop an app that predicts obesity levels at age five using only the few inputs required for the proposed ML models. The intention is to provide an easy-to-use tool for doctors and parents that predicts obesity with good accuracy so that they can make necessary interventions early for children who are at risk of obesity. In conclusion, our assessment on predicting the obesity category of children can provide valuable insight and aid healthcare professionals in taking interventions to help children avoid the life-threatening disease of obesity.

## Figures and Tables

**Table 1:** Dataset size, dataset type, and the input features of different application scenarios.

Application Scenario	Type of Dataset? (Balanced/Unbalanced)	Total Number of Subjects	Number of Subjects in Training Dataset	Number of Subjects in Testing Dataset	Input Features
Single well-child visit	Unbalanced	2039	1427	612	Birth height, weight and BMI, BMI from a single well-child visit, gestational age, gender
Early well-child visit	Balanced	450	315	135	Birth BMI and BMI from different well-child visits up to 2 years of age
Early well-child visit	Unbalanced	224	157	67	Birth BMI and BMI from different well-child visits up to 2 years of age
Multiple random well-child visit	Balanced	450	315	135	Birth BMI and BMI from different multiple random well-child visits
Multiple random well-child visit	Unbalanced	224	157	67	Birth BMI and BMI from different multiple random well-child visits

**Table 2:** Performance of different classification algorithms based on a single well-child visit.

	Categories	Normal	Overweight	Obese
LR	Overall Accuracy	59%		
	Precision	57%	60%	60%
	Recall	63%	4%	84%
	F1 Score	60%	8%	70%
SVM	Overall Accuracy	63%		
	Precision	61%	61%	64%
	Recall	58%	32%	81%
	F1 Score	60%	42%	72%
RF	Overall Accuracy	89%		
	Precision	93%	98%	84%
	Recall	87%	76%	97%
	F1 Score	90%	86%	90%
ANN	Overall Accuracy	61%		
	Precision	59%	52%	64%
	Recall	58%	29%	79%
	F1 Score	58%	37%	71%
*k*-NN	Overall Accuracy	66%		
	Precision	57%	64%	72%
	Recall	67%	49%	74%
	F1 Score	62%	56%	73%
*k*-Means Clustering	Overall Accuracy	31%		
	Precision	27%	24%	51%
	Recall	29%	46%	25%
	F1 Score	28%	31%	34%

**Table 3:** Confusion matrix for random forest implementation for determining obesity category based on a single visit.

	Actual Labels			
		Normal	Overweight	Obese
Predicted Labels	Normal	144	3	8
	Overweight	0	112	2
	Obese	22	32	289

**Table 4:** Performance of random forest classification algorithm for different age groups.

DSB Range	Overall Accuracy	Range Accuracy
0–30	89%	77%
31–90		89%
91–180		92%
181–270		95%
271–365		84%
366–550		88%
551–730		90%
731–910		84%
911–1095		100%
1096–1280		90%
1281–1460		100%
1461–1645		91%
1646–1825		96%

**Table 5:** Results to show the consistency of random forest’s performance.

	Overall Accuracy	Normal		Overweight		Obese	
		Precision	Recall	Precision	Recall	Precision	Recall
Replication 1	87%	86%	83%	90%	77%	86%	93%
Replication 2	89%	88%	90%	92%	80%	89%	93%
Replication 3	92%	91%	93%	91%	87%	93%	93%
Replication 4	92%	91%	90%	93%	86%	91%	95%
Replication 5	93%	95%	92%	92%	86%	92%	96%
Replication 6	90%	92%	90%	93%	80%	88%	95%
Replication 7	91%	94%	90%	95%	81%	87%	95%
Replication 8	92%	90%	91%	93%	86%	92%	94%
Replication 9	91%	91%	90%	92%	84%	91%	95%
Replication 10	89%	93%	87%	98%	76%	84%	97%
Average	91%	91%	90%	93%	82%	89%	95%

**Table 6:** Performance evaluations of ML techniques for determining obesity category based on early visits on an unbalanced dataset.

	Categories	Normal	Overweight	Obese
LR	Overall Accuracy	53%		
	Precision	67%	0%	51%
	Recall	31%	0%	95%
	F1 Score	42%	0%	67%
SVM	Overall Accuracy	44%		
	Precision	40%	0%	47%
	Recall	31%	0%	76%
	F1 Score	35%	0%	58%
RF	Overall Accuracy	69%		
	Precision	67%	67%	71%
	Recall	77%	36%	81%
	F1 Score	71%	47%	76%
ANN	Overall Accuracy	49%		
	Precision	100%	0%	48%
	Recall	8%	0%	100%
	F1 Score	14%	0%	65%
*k*-NN	Overall Accuracy	56%		
	Precision	53%	0%	58%
	Recall	77%	0%	71%
	F1 Score	62%	0%	64%
*k*-Means Clustering	Overall Accuracy	33%		
	Precision	32%	0%	100%
	Recall	100%	0%	4%
	F1 Score	48%	0%	8%

**Table 7:** Performance evaluations of ML techniques for determining obesity category based on early visits on a balanced dataset.

	Categories	Normal	Overweight	Obese
LR	Overall Accuracy	44%		
	Precision	45%	40%	46%
	Recall	38%	44%	51%
	F1 Score	41%	42%	48%
SVM	Overall Accuracy	68%		
	Precision	69%	59%	77%
	Recall	64%	62%	80%
	F1 Score	66%	70%	79%
RF	Overall Accuracy	76%		
	Precision	75%	74%	80%
	Recall	75%	74%	80%
	F1 Score	75%	74%	80%
ANN	Overall Accuracy	56%		
	Precision	58%	49%	62%
	Recall	40%	56%	78%
	F1 Score	47%	52%	69%
*k*-NN	Overall Accuracy	39%		
	Precision	41%	14%	42%
	Recall	53%	5%	51%
	F1 Score	46%	8%	46%
*k*-Means Clustering	Overall Accuracy	29%		
	Precision	15%	32%	30%
	Recall	5%	54%	37%
	F1 Score	8%	40%	33%

**Table 8:** Confusion matrix for random forest implementation for determining obesity category based on early visits (balanced data).

	Actual Labels			
		Normal	Overweight	Obese
Predicted Labels	Normal	40	9	2
	Overweight	8	29	6
	Obese	5	1	35

**Table 9:** Evaluation of performance for obesity prediction based on multiple random visits on an unbalanced dataset.

	Categories	Normal	Overweight	Obese
LR	Overall Accuracy	59%		
	Precision	57%	60%	60%
	Recall	63%	4%	84%
	F1 Score	60%	8%	70%
SVM	Overall Accuracy	63%		
	Precision	61%	61%	64%
	Recall	58%	32%	81%
	F1 Score	60%	42%	72%
RF	Overall Accuracy	89%		
	Precision	93%	98%	84%
	Recall	87%	76%	97%
	F1 Score	90%	86%	90%
ANN	Overall Accuracy	61%		
	Precision	59%	52%	64%
	Recall	58%	29%	79%
	F1 Score	58%	37%	71%
*k*-NN	Overall Accuracy	66%		
	Precision	57%	64%	72%
	Recall	67%	49%	74%
	F1 Score	62%	56%	73%
*k*-Means Clustering	Overall Accuracy	31%		
	Precision	27%	24%	51%
	Recall	29%	46%	25%
	F1 Score	28%	31%	34%

**Table 10:** Evaluation of performance for obesity prediction based on multiple random visits on a balanced dataset.

	Categories	Normal	Overweight	Obese
LR	Overall Accuracy	56%		
	Precision	54%	43%	70%
	Recall	45%	56%	63%
	F1 Score	49%	49%	67%
SVM	Overall Accuracy	62%		
	Precision	58%	44%	83%
	Recall	57%	51%	75%
	F1 Score	57%	48%	79%
RF	Overall Accuracy	64%		
	Precision	57%	47%	87%
	Recall	52%	59%	77%
	F1 Score	87%	77%	82%
ANN	Overall Accuracy	44%		
	Precision	48%	33%	64%
	Recall	32%	62%	40%
	F1 Score	38%	43%	49%
*k*-NN	Overall Accuracy	56%		
	Precision	51%	39%	89%
	Recall	48%	56%	63%
	F1 Score	49%	46%	74%
*k*-Means Clustering	Overall Accuracy	54%		
	Precision	49%	41%	93%
	Recall	41%	74%	50%
	F1 Score	44%	53%	65%

**Table 11:** Confusion matrix for random forest implementation for determining obesity category based on multiple random well-child visits (unbalanced data).

	Actual Labels			
		Normal	Overweight	Obese
Predicted Labels	Normal	16	1	0
	Overweight	2	12	2
	Obese	0	2	32

## Data Availability

Not applicable.
